# Structural and Magnetic Phase Transitions in BiFe_1 − x_Mn_x_O_3_ Solid Solution Driven by Temperature

**DOI:** 10.3390/nano12091565

**Published:** 2022-05-05

**Authors:** Dmitry V. Karpinsky, Maxim V. Silibin, Siarhei I. Latushka, Dmitry V. Zhaludkevich, Vadim V. Sikolenko, Hanan Al-Ghamdi, Aljawhara H. Almuqrin, M. I. Sayyed, Alexei A. Belik

**Affiliations:** 1 Scientific-Practical Materials Research Centre of NAS of Belarus, 220072 Minsk, Belarus; latushkasi@gmail.com (S.I.L.); geludkevichdima@mail.ru (D.V.Z.); 2 Institute of Advanced Materials and Technologies, National Research University of Electronic Technology “MIET”, Zelenograd, 124498 Moscow, Russia; sil_m@mail.ru (M.V.S.); sikolen@jinr.ru (V.V.S.); 3 Frank Laboratory of Neutron Physics, Joint Institute for Nuclear Research, 141980 Dubna, Russia; 4 Division Technical Petrophysics, Institute of Applied Geosciences, Karlsruhe Institute of Technology, 76131 Karlsruhe, Germany; 5 Department of Physics, College of Science, Princess Nourah Bint Abdulrahman University, P.O. Box 84428, Riyadh 11671, Saudi Arabia; hmalghmdi@pnu.edu.sa (H.A.-G.); ahalmoqren@pnu.edu.sa (A.H.A.); 6 Department of Physics, Faculty of Science, Isra University, Amman 11622, Jordan; dr.mabualssayed@gmail.com; 7 Department of Nuclear Medicine Research, Institute for Research and Medical Consultations (IRMC), Imam Abdulrahman Bin Faisal University (IAU), Dammam 31441, Saudi Arabia; 8 International Center for Materials Nanoarchitectonics (WPI-MANA), National Institute for Materials Science (NIMS), Namiki 1-1, Tsukuba, Ibaraki 305-0044, Japan; alexei.belik@nims.go.jp

**Keywords:** crystal structure, magnetic state, multiferroics, phase transitions, magnetometry, X-ray diffraction, synchrotron diffraction

## Abstract

The crystal structure and magnetic state of the (1 − x)BiFeO_3_-(x)BiMnO_3_ solid solution has been analyzed by X-ray diffraction using lab-based and synchrotron radiation facilities, magnetization measurements, differential thermal analysis, and differential scanning calorimetry. Dopant concentration increases lead to the room-temperature structural transitions from the polar-active rhombohedral phase to the antipolar orthorhombic phase, and then to the monoclinic phase accompanied by the formation of two-phase regions consisting of the adjacent structural phases in the concentration ranges 0.25 < x_1_ < 0.30 and 0.50 ≤ x_2_ < 0.65, respectively. The accompanied changes in the magnetic structure refer to the magnetic transitions from the modulated antiferromagnetic structure to the non-colinear antiferromagnetic structure, and then to the orbitally ordered ferromagnetic structure. The compounds with a two-phase structural state at room temperature are characterized by irreversible temperature-driven structural transitions, which favor the stabilization of high-temperature structural phases. The magnetic structure of the compounds also exhibits an irreversible temperature-induced transition, resulting in an increase of the contribution from the magnetic phase associated with the high-temperature structural phase. The relationship between the structural parameters and the magnetic state of the compounds with a metastable structure is studied and discussed depending on the chemical composition and heating prehistory.

## 1. Introduction

Functional oxides of transition metal ions possessing multiferroic properties have attracted the interest of researchers and have been studied during recent decades. The widely studied bismuth ferrite is a single-phase multiferroic with high temperatures of ferroelectric (1100 K) and magnetic (650 K) transitions [1,2,3]. Hence, the magnetic structure of bismuth ferrite is modulated *G*-type antiferromagnetic, and it can be disrupted either by a strong magnetic field [2,4] or by chemical substitution [5,6], thus leading to the stabilization of spontaneous magnetization at room temperature and a release of the linear magnetoelectric effect [7,8]. It is known that bismuth manganite is a magnetoelectric material with a perovskite structure, displaying monoclinic distortion of the unit cell, and the temperature of the magnetic transition, T_C_, is ~100 K. The ferromagnetic state of BiMnO_3_ is caused by orbital ordering of Mn^3+^ ions, which are stable up to ~475 K, wherein the type of magnetic coupling strongly depends on the chemical bond lengths, Mn–O–Mn, and the related bond angles, and can be modified by chemical doping or other factors. Above T ~ 770 K, there is a phase transition to the orthorhombic structure, described by the space group *Pnma* [9].

It is known that bismuth ferrite (BiFeO_3_) and bismuth manganite (BiMnO_3_) can form a solid solution in the entire composition range [10], while Mn-rich compounds can be prepared only by the high-pressure high-temperature (HPHT) synthesis method [2,11]. The crystal structure and magnetic state of the solid solution (1 − x)BiFeO_3_-(x)BiMnO_3_ strongly depends on the dopant content [12]. Thus, the crystal structure of the compounds changes from the polar rhombohedral single phasic specific for the initial BiFeO_3_ to the antipolar orthorhombic, and then to the monoclinic one (BiMnO_3_) via a stabilization of the respective two-phase regions. Chemical substitution of Mn^3+^ ions for Fe^3+^ ions in BiFeO_3_ causes a decrease in the temperature of the magnetic transition [13], which is accompanied by a transformation of the magnetic state from the antiferromagnetic to the long-range ferromagnetic one. Thus, the magnetic structure of the compounds changes upon the chemical substitution from the *G*-type antiferromagnetic one (BiFeO_3_), having spiral modulation, to the ferromagnetic structure, caused by the ordering of Mn d_z_^2^ orbitals (BiMnO_3_) [14,15].

Recently, it was noted that compounds of the solid solution (1 − x)BiFeO_3_-(x)BiMnO_3_ (0.2 < x < 0.5) are characterized by irreversible behavior of magnetization after being subjected to the external magnetic field above the magnetic transition temperature [16], and the authors suggested an “extrinsic” origin of the metastable magnetic state related to chemical inhomogeneities of the samples. The diffraction data also point to a metastable structural state of the compounds with a two-phase crystal structure at room temperature, i.e., the ratio of the coexisting phases is notably changed in the compounds subjected to temperature increases up to ~400–500 °C. The above-mentioned facts confirm a strong correlation between the structural transformation and the changes in the magnetic state of the compounds (1 − x)BiFeO_3_-(x)BiMnO_3_, while the available data are not enough to itemize an evolution of the magnetic structure depending on the structural parameters.

In the present study, the crystal structure and magnetic properties of the compounds BiFe_1 − x_Mn_x_O_3_ have been investigated depending on the chemical composition and heating prehistory. The obtained results have allowed to reveal an origin of the magnetic state transformation as well as to correlate it with the changes that occurred in the structural state of the compounds depending on their chemical composition and heating prehistory.

## 2. Materials and Methods

Ceramic compounds (1 − x)BiFeO_3_-(x)BiMnO_3_ were prepared from the mixtures of simple oxides Bi_2_O_3_, Fe_2_O_3_, and Mn_2_O_3_ in a stoichiometric ratio using the conventional ceramic method (x < 0.5) [17] and the high-pressure high-temperature (HPHT) method (0.5 ≤ x ≤ 1) [10]. The HPHT synthesis was implemented utilizing a high-pressure device under a pressure of ~5 GPa, with a simultaneously applied temperature of 1600 K during 40 min, and the samples were sealed in platinum containers. After their synthesis, the samples were cooled down to room temperature with a simultaneous gradual decrease of pressure. The above-mentioned synthesis methods and conditions allowed to prepare ceramics with an average crystallite size of about a few hundred nanometers. Crystal structures of the ceramics were determined based on the diffraction results obtained by laboratory equipment (PanAlytical X’pert Pro diffractometer, Kraków, Poland, λ = 1.5406 Å), as well as synchrotron powder diffraction (KMC-2 instrument at BESSY-II, Berlin, Germany, λ = 1.5405 Å [18], and BL02B2 instrument at SPring-8, Osaka, Japan, λ = 0.4202 Å) in the temperature interval 300–900 K, with a heating rate of about 100 K per hour (samples were cooled in a furnace down to room temperature). The diffraction data were examined using FullProf software (Rietveld method) [19]. Magnetization measurements were performed using MPMS (Cryogenic Ltd., London, UK). Differential scanning calorimetry measurements and differential thermal analysis were performed using the Netzsch 204 F1 Phoenix setup in nitrogen gas with a heating/cooling rate of 10 K per min.

## 3. Results

Examination of the X-ray diffraction patterns obtained by the compounds BiFe_1 − x_Mn_x_O_3_ by laboratory and synchrotron techniques confirmed the changes of the structure from the rhombohedral (sp. gr. *R3c*) to the orthorhombic (*Pnma*) and then to the monoclinic structure (*C2/c*) that occurred with the increase in the dopant concentration, which is in agreement with the results available in a previously published paper [10]. The declared sequence of the structural transitions was complemented by a gradual modification of the unit call parameters (Supplementary Materials, Figures S1–S4), leading to a decrease in the volume of the unit cell, and thus points at the formation of a continuous solid solution in the whole concentration range. Optimization of the synthesis method allowed to narrow the two-phase concentration region down to ~5 mol.%, where both the orthorhombic phase and the rhombohedral phase coexist, and to get rid of the impurity phases specific for this solid solution [12,20]. An increase of the concentration of the dopant ions up to 25 mol.% led to the stabilization of the orthorhombic phase (sp. gr. *Pnma*), described by the metric √2a_p_…4a_p_…2√2a_p_ (a_p_—primitive perovskite parameter), which is four times larger than that of the conventional orthorhombic cell specific for orthoferrites with a perovskite structure [21,22]. The relatively large unit cell parameters are caused by additional distortion of the lattice because of the antipolar ordering of the dipole moments formed along the *c*-axis, in contrast to the polar order specific for the initial compound BiFeO_3_. The compound with x = 0.3 is characterized by the structural state with two phases, with the dominant orthorhombic phase and a minor amount (~10 mol. %) of the rhombohedral phase (the phases ratio, structural parameters, coordinates, unit cell volumes, etc., refined by the Rietveld method, are provided in the Supplementary Materials, Table S1). In the concentration range specific for the single-phase orthorhombic structure, the unit cell parameters changed in a different way, showing a decrease in the *a*- and *c*-parameters, whereas the magnitude of the *b*-parameter increased, which reflects a strengthening of antipolar distortion and enlargement of the related dipole moments. A stabilization of the monoclinic phase was observed in the concentration range of 0.5 ≤ x < 0.65, which was accompanied by a slight decrease in the volume of the unit cell.

**Figure 1:**
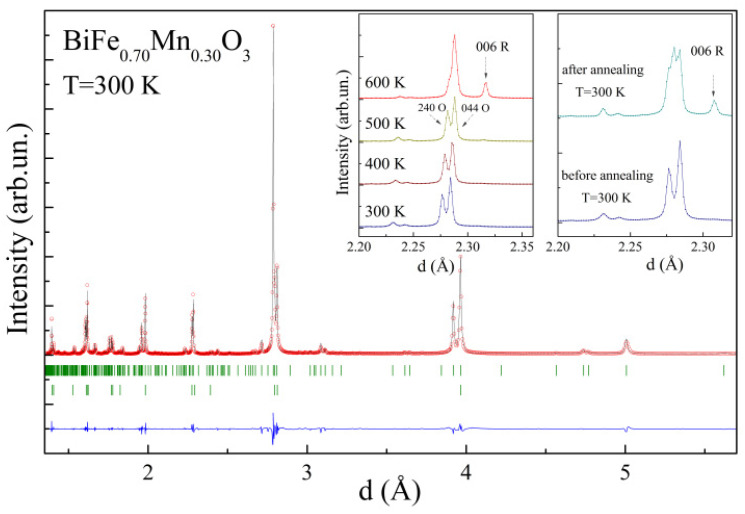
Room-temperature synchrotron diffraction pattern of BiFe_0.7_Mn_0.3_O_3_. The left inset shows temperature-driven changes of the reflections attributed to the different structural phases (O—*Pnma* phase; R—*R3c* phase), and the right inset shows specific reflections at room temperature for the compound before and after annealing at ~650 K.

**Figure 2:**
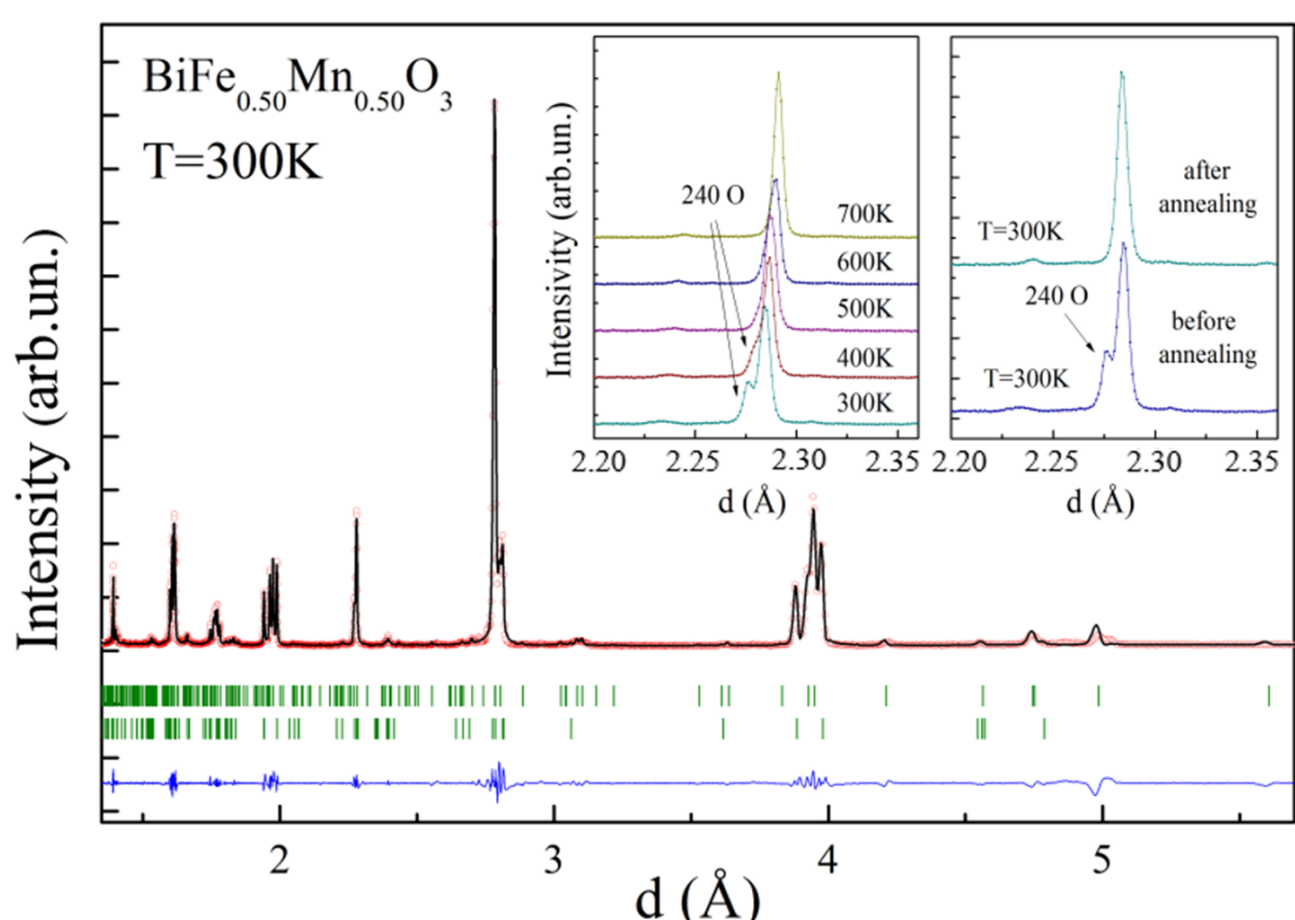
Room-temperature synchrotron diffraction pattern of BiMn_0.5_Fe_0.5_O_3_ with a two-phase structural state (upper vertical dashes denote Bragg positions specific for the orthorhombic phase, bottom vertical dashes denote the monoclinic phase). The left inset shows temperature-dependent changes of the reflections attributed to the dominant orthorhombic phase, and the right inset shows specific reflections before and after annealing of the compound at T ~ 700 K (O—*Pnma* phase).

The unit cell metric notably changed and became √6a_p_…√2a_p_…√6a_p_, thus leading to a rearrangement in the dipole moments as well as the formation of a new type of orientation of the magnetic moments. The temperature increase led to drastic modification of the compounds’ structure over the entire range of the solid solution. The rhombohedral compounds, i.e., those having an Mn content less than 25 mol.%, showed temperature-induced phase transition similar to that observed for the initial compound BiFeO_3_, i.e., from the polar-active rhombohedral phase to the orthorhombic phase with a non-polar character [12,23]. The solid solutions characterized by dominant orthorhombic or monoclinic structures at room temperature showed a non-monotonous evolution of the structure driven by temperature. The temperature-dependent diffraction patterns recorded for the compound with x = 0.3, with a dominant orthorhombic phase at room temperature above a temperature of T ~ 500 K, showed a notable increase in the intensity of the reflections specific for the rhombohedral phase (Figure 1). The amount of the rhombohedral phase increased at the expense of the orthorhombic phase, but this structural transition was not completed up to a temperature of ~750 K, and above this temperature, a chemical decomposition began. It should be noted that room-temperature diffraction data obtained for the compound with x = 0.3 before and after heating the sample up to 650 K showed notable differences, pointing to an increased amount of the rhombohedral phase, as seen by a rapid increase in the intensity of the reflection 006_R_ specific for the rhombohedral phase (Figure 1, inset). The estimated phase ratio, O:R, was 9:1 and 5:1, before and after annealing, respectively (see the Supplementary Materials, Table S2). The scenario of the irreversible phase transition is also supported by the increased magnitude of the average unit cell volume observed for the compound after annealing. Temperature-dependent diffraction measurements of the compound with x = 0.5, with a dominant orthorhombic structural state and a minor amount of the monoclinic phase at room temperature, showed even more pronounced irreversible structural transformations. In particular, the diffraction patterns at elevated temperatures showed a drastic decrease in the orthorhombic distortion, thus leading to a stabilization of cubic-like phase, as declared in [10], while this transition was not completed up to an onset of the chemical decomposition. The room-temperature pattern recorded for the sample subjected to heating up to 700 K showed a stabilization of the two-phase structural state with a dominant cubic-like phase (Figure 2, inset).

**Figure 3:**
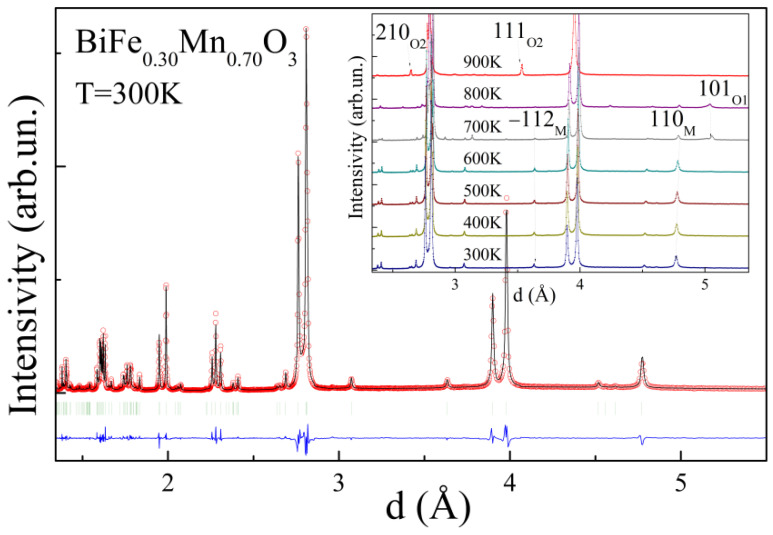
Room-temperature synchrotron diffraction pattern of BiFe_0.3_Mn_0.7_O_3_. The inset shows temperature evolution of the reflections attributed to the monoclinic and the orthorhombic phases (M—*C2/c* phase; O_2_—*Pnma* phase with metric √2a_p_·4a_p_·2√2a_p_, a_p_—primitive perovskite unit cell parameter).

**Figure 4:**
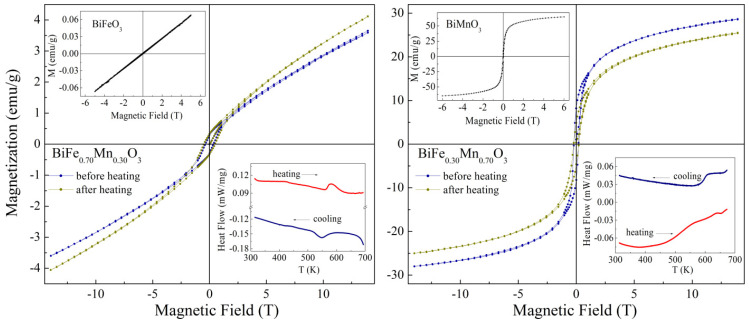
Isothermal-dependent magnetization curves of the compounds BiFe_1 − x_Mn_x_O_3_ (x = 0.3, 0.7) recorded at temperature T = 5 K before and after annealing at T ~ 700 K. The insets show DSC curves of the compounds and field dependence of magnetization recorded at T = 5 K for the extreme compounds BiFeO_3_ and BiMnO_3_.

The compound with x = 0.7 is single phasic with a monoclinic structure, and at room temperature, the diffraction patterns were successfully refined using the sp. gr. *C2/c,* which is specific for the extreme compound BiMnO_3_ [14,24] (see the Supplementary Materials, Table S3). Temperature-dependent diffraction data as well as DSC results recorded for the compound with x = 0.7, i.e., with a single-phase monoclinic structure, point at the phase transition to the antipolar orthorhombic state at temperatures above 650 K (Figure 3 and Figure 4, insets). The two-phase structural state remained stable up to 850 K, and in the narrow temperature range of 820–870 K, the compound became single phasic with an orthorhombic structure. At temperatures above 870 K, a new non-polar orthorhombic phase stabilized, and the compound remained single-phase orthorhombic up to the temperature of chemical decomposition of ~950 K (Figure 3). The room-temperature pattern recorded for the compound after heating up to 600 K showed a stabilization of the two-phase structural state with nearly equal amounts of the monoclinic and the antipolar orthorhombic phases.

The magnetic structure of the compounds also notably changed with the chemical doping, and one can assume three different concentration regions ascribed to the different magnetic states. The compounds with rhombohedral phase are characterized by a spatially modulated spin structure specific for the initial BiFeO_3_ [25]. Increases in the concentration of Mn ions led to a disruption of the modulated magnetic structure formed by Fe^3+^ ions, thus leading to a stabilization of the non-colinear antiferromagnetic structure accompanied by an appearance of the non-zero remanent magnetization in the solid solutions with 0.3 < x < 0.5. The changes in the magnetic structure of the doped compounds as compared to the initial BiFeO_3_ were evidently observed by the results of magnetization measurements (Figure 4). Further increases in the Mn content caused a frustration of the long-range antiferromagnetic structure, and the compounds within the concentration range 0.4 < x < 0.6 were characterized by the formation of a short-range magnetic order, as confirmed by magnetometry data. The compounds with Mn-rich chemical compositions (x > 0.6) were characterized by a notable increase in the low-temperature magnetization, thus denoting the formation of a new type of magnetic structure ascribed to the ferromagnetic state. The results of isothermal magnetization measurements of the compound with x = 0.7 showed a distinct hysteresis-like loop at a temperature of 5 K (Figure 4), while a long-range ferromagnetic order was not formed.

Taking into account the results obtained by the diffraction techniques, as well as magnetometry measurements, one can conclude that there was a strong correlation between the type of magnetic structure and the structural state of the solid solutions. The reduction in the magnetization value detected for the compound with x = 0.7 subjected to annealing at a high temperature was associated with a decrease in the amount of the monoclinic phase characterized by the long-range ferromagnetic structure (Figure 4), as compared to the weak ferromagnetic or frustrated magnetic order specific for the orthorhombic phase. A slight increase in the value of remanent magnetization was also observed for the compound with x = 0.3 after annealing, which was also caused by a change in the ratio of the coexisting structural phases. In particular, an increase in the amount of the rhombohedral phase, from ~10 mol.% before annealing up to ~25 mol.% after annealing, caused related changes in the magnetic state of the compound with x = 0.3 (Figure 4).

**Figure 5:**
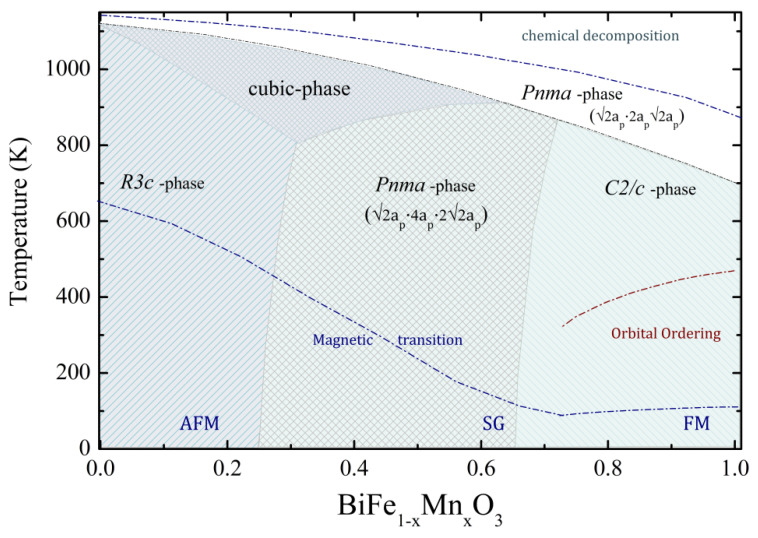
Preliminary structural and magnetic phase diagram of the solid solution BiMnO_3_-BiFeO_3_ depending on temperature and chemical composition.

The results of the diffraction measurements as well as the data obtained by DSC/DTA methods and magnetometry obtained for the solid solutions BiMnO_3_-BiFeO_3_ have allowed the construction of the preliminary temperature (T)–composition (*x*) phase diagram (Figure 5). The T*–x* diagram denotes the dopant concentration and temperature ranges of the structural and magnetic phases ascribed to the compounds with different crystal symmetry and magnetic structures. The dopant concentration-induced structural transition from the polar rhombohedral phase to the antipolar orthorhombic phase observed for the compounds at room temperature was observed in the concentration range 0.25 < x_1_ < 0.30, and the phase boundary corresponding to the coexisting orthorhombic and monoclinic phases was located in the concentration range 0.50 ≤ x_2_ < 0.65. One should note that high-temperature transition to the non-polar orthorhombic phase is considered to have an extrinsic character, as it occurred at temperatures close to the chemical decomposition and was most probably triggered by a release of oxygen ions [12,23]. Heating of the samples below the temperature of the chemical degradation led to the irreversible phase transition, affecting both the structural and magnetic state of the compounds.

## 4. Conclusions

Analysis of the diffraction measurements data along with the results of magnetometry and DSC/DTA experiments allowed for clarifying the background of temperature- and concentration-driven structural and magnetic phase transitions in the solid solutions BiFe_1 − x_Mn_x_O_3_. It was shown that the dopant concentration increase caused the phase transition from the rhombohedral to the orthorhombic structure, leading to a disruption of the modulated antiferromagnetic structure and the formation of the non-colinear antiferromagnetic structure associated with weak ferromagnetism. A further increase of the concentration of Mn ions led to the formation of an orbitally ordered ferromagnetic structure. Annealing of the compounds at high temperature (~700 K) caused irreversible transitions in the structural state of the compounds, thus leading to stabilization of the crystal structure specific for the high-temperature range. The magnetic states of the compounds significantly changed after thermal annealing, and the transformation of the magnetic state was caused by an increase in the amount of the appropriate structural phase. Thus, in the Fe-rich compounds, the changes in the magnetic state were associated with an increase of the rhombohedral phase characterized by weak ferromagnetism, while the magnetic state of the Mn-rich compounds was determined by an increased amount of the orthorhombic phase with a frustrated magnetic order.

## Data Availability

Data is contained within the article or supplementary material.

## Supplementary Materials

The following supporting information can be downloaded at: https://www.mdpi.com/article/10.3390/nano12091565/s1, Figure S1: Visualization of the unit cells: the antipolar orthorhombic (O-phase, left image) and the rhombohedral one (R-phase, right image). Figure S2. DSC data for compound BiFe_0.70_Mn_0.30_O_3_. Figure S3: Visualization of the unit cells: the antipolar orthorhombic (O-phase, left image) and the mono-clinic one (M-phase, right image). Figure S4: Visualization of the monoclinic unit cell (M-phase). Table S1: Visualization of the monoclinic unit cell (M-phase). Table S2: BiFe_0.50_Mn_0.50_O_3_ (as-prepared sample, SPD data refined at T = 300 K). Table S3: BiFe_0.30_Mn_0.70_O_3_ (as-prepared sample, SPD data refined at T = 300 K).
