# Supplementary material for: Structural and Magnetic Phase Transitions in BiFe1 − xMnxO3 Solid Solution Driven by Temperature
Source: Nanomaterials (Basel). 2022 May 5;12(9):1565. doi: 10.3390/nano12091565 (PMC9103236; doi:10.3390/nano12091565)
Supplement: Supplementary file 1 [file nanomaterials-12-01565-s001.zip › nanomaterials-1680008-supplementary.pdf]

Supplementary Materials

Structural and magnetic phase transitions in $BiFe_{1-x}Mn_xO_3$ solid solution driven by temperature

Dmitry.**V. Karpinsky** [1,2,*], **Maxim V. Silibin** [2], **Siarhei I. Latushka** [1,2], **Dmitry V. Zhaludkevich** [1,2], **Vadim V. Sikolenko** [2,3,4], **Hanan Al-Ghamdi** [5], **Aljawhara H. Almuqrin** [5], **M. I. Sayyed** [6,7] and **Alexei A. Belik** [8]

[1] Scientific-Practical Materials Research Centre of NAS of Belarus, 220072 Minsk, Belarus; latushkasi@gmail.com (S.I.L.); geludkevichdima@mail.ru (D.V.Z.)
[2] Institute of Advanced Materials and Technologies, National Research University of Electronic Technology "MIET", 124498 Zelenograd, Moscow, Russia; sil_m@mail.ru (M.V.S.); sikolen@jinr.ru (V.V.S.)
[3] Frank Laboratory of Neutron Physics, Joint Institute for Nuclear Research Joint Institute for Nuclear Research, 141980 Dubna, Russia
[4] Division Technical Petrophysics, Institute of Applied Geosciences, Karlsruhe Institute of Technology, 76131 Karlsruhe, Germany
[5] Department of Physics, College of Science, princess Nourah bint Abdulrahman University, 84428, Riyadh 11671, Saudi Arabia; hmalghmdi@pnu.edu.sa (H.A.-G.); ahalmoqren@pnu.edu.sa (A.H.-A.)
[6] Department of Physics, Faculty of Science, Isra University, Amman, Jordan; dr.mabualssayed@gmail.com
[7] Department of Nuclear Medicine Research, Institute for Research and Medical Consultations (IRMC), Imam Abdulrahman Bin Faisal University (IAU), Dammam, 31441, Saudi Arabia
[8] International Center for Materials Nanoarchitectonics (WPI-MANA), National Institute for Materials Science (NIMS), Namiki 1-1, Tsukuba, Ibaraki, 305−0044, Japan; alexei.belik@nims.go.jp
* Correspondence: dmitry.karpinsky@gmail.com

Table S1. $BiFe_{0.70}Mn_{0.30}O_3$ (as-prepared sample, SPD data refined at T = 300 K).

**O-phase (~90%): Pnma space group; a = 5.5937 Å, b = 15.6785 Å, c = 11.2428 Å, Vol. = 986.00 Å^3 (~61.63 Å^3 per prim.)**					**R-phase (~10%): R3c space group; a = 5.6627 Å, c = 13.2543 Å, Vol. = 368.08 Å^3 (~61.34 Å^3 per prim.)**				
**atom**	**site**	**x**	**y**	**z**	**atom**	**site**	**x**	**y**	**z**
Bi1	8d	0.21755	0.01031	0.62373	Bi	6a	0	0	0
Bi2	4c	0.19322	1/4	0.62478	Fe|Mn	6a	0	0	0.23053
Bi3	4c	0.78535	1/4	0.36228	O1	18b	0.42840	0.01486	0.95264
Fe|Mn1	8d	0.22848	0.12268	0.87476					
Fe|Mn2	8d	0.26247	0.62444	0.88065					
O1	8d	0.85862	0.00636	0.63359					
O2	4c	0.77883	1/4	0.59362					
O3	4c	0.18536	1/4	0.42279					
O4	8d	0.04437	0.11297	0.72374					
O5	8d	0.02537	0.63780	0.75148					
O6	8d	0.00000	0.39371	0.50000					
O7	8d	0.00000	0.37667	0.00000					

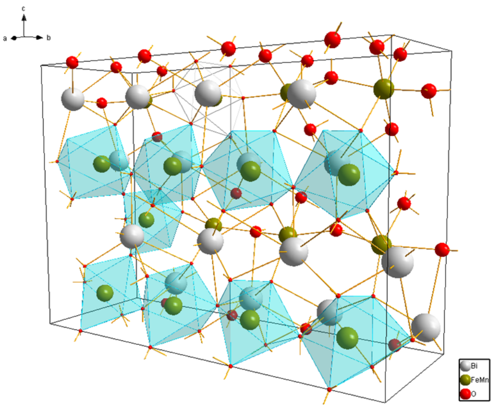

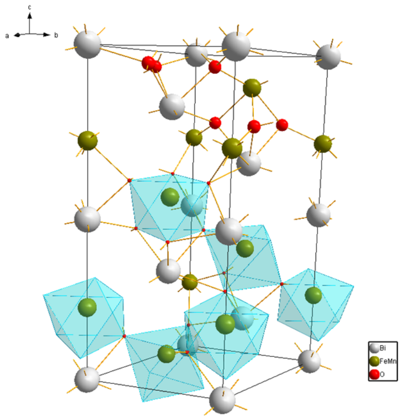

Figure S1. Visualization of the unit cells: the antipolar orthorhombic (O-phase, left image) and the rhombohedral one (R-phase, right image).

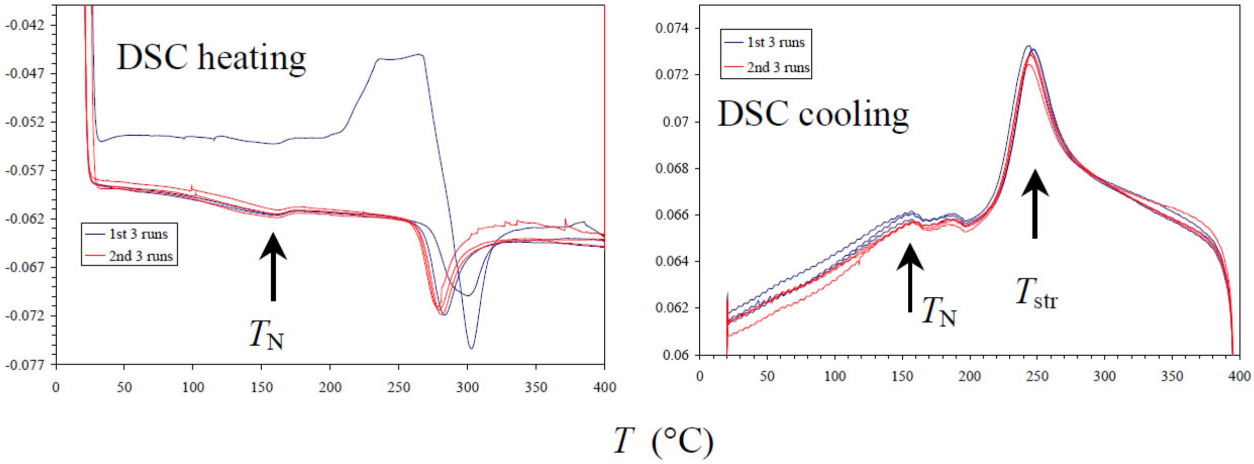

Figure S2. DSC data for compound $BiFe_{0.70}Mn_{0.30}O_3$.

Table S2. $BiFe_{0.50}Mn_{0.50}O_3$ (as-prepared sample, SPD data refined at T = 300 K).

**O-phase (~66%): Pnma space group; a = 5.5677 Å, b = 15.7104 Å, c = 11.2146 Å, Vol. = 980.96 Å^3 (~61.31 Å^3 per prim.)**				
**atom**	**site**	x	y	z
Bi1	8d	0.21292	0.01251	0.61724
Bi2	4c	0.23916	1/4	0.62418
Bi3	4c	0.77430	1/4	0.37469
Fe|Mn1	8d	0.26408	0.11082	0.84479
Fe|Mn2	8d	0.26766	0.62685	0.88304
O1	8d	0.88963	0.00375	0.69238
O2	4c	0.69420	1/4	0.60875
O3	4c	0.10489	1/4	0.46343
O4	8d	0.02852	0.17378	0.71291
O5	8d	0.05771	0.77304	0.82047
O6	8d	0.00000	0.38177	0.50000
O7	8d	0.00000	0.39019	0.00000

**M-phase (~33%): C2/ c space group; a = 9.5984 Å, b = 5.6341 Å, c = 9.6033 Å, β = 108.21, Vol. = 493.34 Å^3 (~61.67 Å^3 per prim.)**				
**atom**	**site**	x	y	z
Bi	8f	0.13205	0.25310	0.13653
Mn|Fe1	4e	0	0.22143	3/4
Mn|Fe2	4d	1/4	1/4	1/2
O1	8f	0.05082	0.28645	0.59208
O2	8f	0.20126	0.58942	0.30602
O3	8f	0.37333	0.48382	0.10537

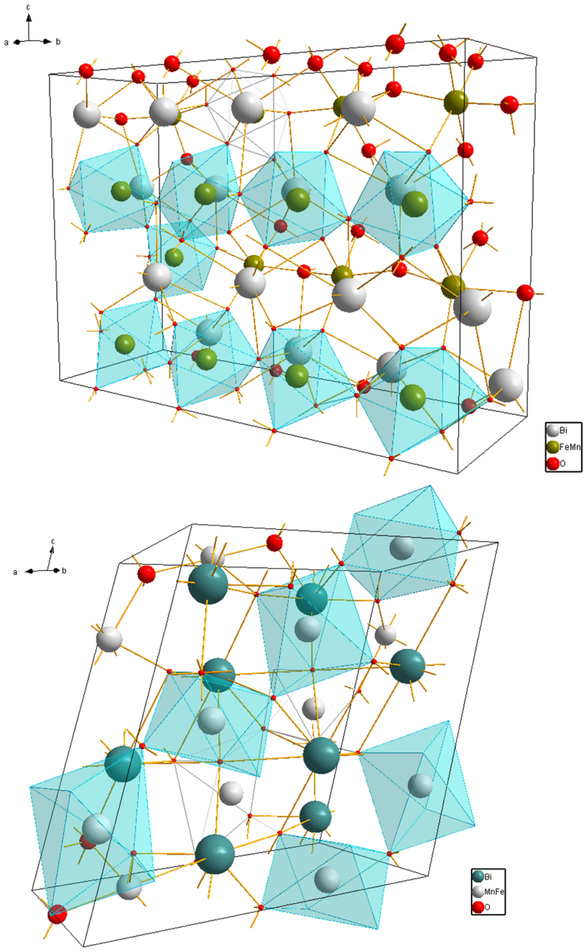

Figure S3. Visualization of the unit cells: the antipolar orthorhombic (O-phase, left image) and the monoclinic one (M-phase, right image).

Table S3. $BiFe_{0.30}Mn_{0.70}O_3$ (as-prepared sample, SPD data refined at T = 300 K).

**M-phase: C2/ c space group; a = 9.5172 Å, b = 5.6208 Å, c = 9.7214 Å, β = 108.2962, Vol. = 493.76 Å^3 (~61.72 Å^3 per prim.)**				
**atom**	**site**	**x**	**y**	z
Bi	8f	0.13609	0.23264	0.12793
Mn|Fe1	4e	0	0.19849	3/4
Mn|Fe2	4d	1/4	1/4	1/2
O1	8f	0.06125	0.20249	0.55167
O2	8f	0.16390	0.56907	0.35677
O3	8f	0.36968	0.52691	0.13010

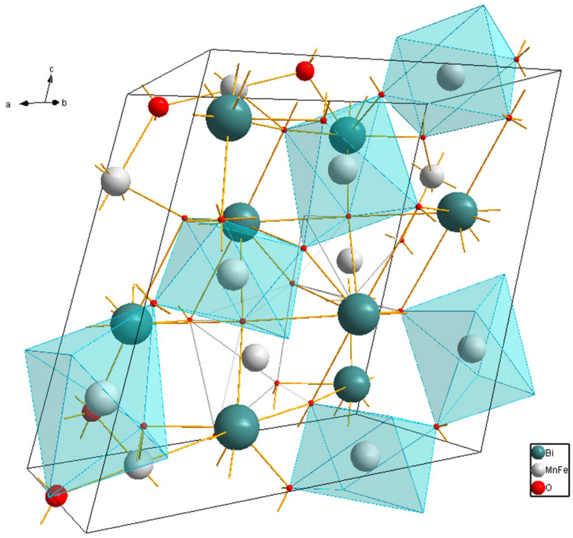

Figure S4. Visualization of the monoclinic unit cell (M-phase).
